# FOXO3a‐mediated long non‐coding RNA LINC00261 resists cardiomyocyte hypoxia/reoxygenation injury via targeting miR23b‐3p/NRF2 axis

**DOI:** 10.1111/jcmm.15292

**Published:** 2020-06-18

**Authors:** Ruining Zhang, Yongjun Li, Xiaopeng Liu, Shan Qin, Bingyan Guo, Liang Chang, Liu Huang, Suyun Liu

**Affiliations:** ^1^ Department of Cardiology The Second Hospital of Hebei Medical University Shijiazhuang HeBei China; ^2^ The Hebei Institute of Cardiovascular and Cerebrovascular Diseases (YL) Shijiazhuang China; ^3^ Department of Neurosurgery The Second Hospital of Hebei Medical University Shijiazhuang China; ^4^ The Graduate School GuiZhou medical university GuiYang China

**Keywords:** apoptosis, FOXO3a, hypoxia, long non‐coding RNA, miR‐23b‐3p, NRF2, reoxygenation

## Abstract

Ischemia/reperfusion (I/R)‐mediated acute myocardial infarction (AMI) is a major pathological factor implicated in the progression of ischemic heart disease (IHD). Long non‐coding RNA plays an important role in regulating the occurrence and development of cardiovascular disease. The aim of this study was to investigate the regulating role of LINC00261 in hypoxia/reoxygenation (H/R)‐induced cardiomyocyte apoptosis. The relative expression of LINC00261, miR‐23b‐3p and NRF2 were determined in rats I/R myocardial tissues and H/R‐induced cardiomyocytes. The rat model and cell model of LINC00261 overexpression were established to investigate the biological function of LINC00261 on H9C2 cell. The interaction between LINC00261, miR‐23b‐3p, NRF2 and FOXO3a was identified using bioinformatics analysis, luciferase reporter assay, RNA immunoprecipitation (RIP) assay, chromatin immunoprecipitation (CHIP) assay and qRT‐PCR. The expression of LINC00261 was significantly down‐regulated in myocardial tissues and H9C2 cell. Overexpression of LINC00261 improves cardiac function and reduces myocardium apoptosis. Interestingly, transcription factor FOXO3a was found to promote LINC00261 transcription. Moreover, LINC00261 was confirmed as a spong of miR23b‐3p and thereby positively regulates NRF2 expression in cardiomyocytes. Our findings reveal a novel role for LINC00261 in regulating H/R cardiomyocyte apoptosis and the potency of the LINC00261/miR‐23b‐3p/NRF2 axis as a therapeutic target for the treatment of MIRI.

## 1 INTRODUCTION

Reperfusion therapy after myocardial infarction (MI), including percutaneous coronary intervention (PCI), and thrombolysis effectively saved the infarcted myocardium. However, the restored blood supply after myocardial ischemia causes progressive damage to some myocardium.^1^ This pathological process is called myocardial ischemia‐reperfusion injury (MIRI). MIRI is a common perioperative complication, causing an increase in mortality, and occurs during coronary bypass surgery and extracorporeal circulation surgery.^2^ During hypoxia, especially the reoxygenation phase, a large increase in oxidative stress and ROS production activates the mitochondrial apoptotic program, triggering the caspase cascade, and eventually causing cardiomyocyte apoptosis.^3^, ^4^ How to reduce MIRI is of great significance for improving the clinical prognosis of patients with myocardial I/R.

Long non‐coding RNAs (LncRNAs) refer to a class of RNA whose transcript is more than 200 nucleotides in length but with little to no protein‐coding capacity. It can regulate gene expression through three pathways: apparent modification, transcription and post‐transcriptional regulation.^5^ LncRNA can be expressed in the cytoplasm and nucleus. The lncRNAs in the cytoplasm can binding to the target messenger RNA (mRNA) promotes or prevents translation, or acts as an endogenous ‘sponge’ for microRNAs, thereby inhibiting the negative regulation of miRNAs on target gene expression. For instance, lncRNA MAGI2‐AS3 has been shown regulated by transcription factor BRD4 and promoted gastric cancer progression by sponging miR‐141/200a.^6^ In H/R‐induced vascular endothelial cell model, lncRNA SNHG1 was identified function as a competing endogenous RNA (ceRNA) of miR‐140‐3p through the HIF‐α/VEGF signalling pathway.^7^

Numerous studies have confirmed that lncRNAs affects myocardial cell necrosis, apoptosis and autophagy by regulating gene expression, and thus participate in the pathological process of MIRI.^8^, ^9^ In addition, lncRNAs have high potential and value as clinical diagnostic markers and therapeutic targets for MI.^10^ Long non‐coding RNA LINC00261 has been shown closely related to the development of multiple tumours, including gastric cancer,^11^ hepatocellular carcinoma.^12^ Study in lung adenocarcinoma (LUAD) cell lines have shown that LINC00261 overexpression obviously inhibits cell proliferation, migration and expression of DNA lesion genes, suggesting that LINC00261 is a tumour suppressor.^13^ However, it is unclear whether LINC00261 plays a regulatory role in MIRI.

In the present study, we explored LINC00261 functional role and mechanism in MIRI. The results demonstrated that FOXO3a‐mediated overexpressing of LINC00261 reduces H/R‐induced cardiomyocytes apoptosis by targeting miR‐23b‐3p/NRF2 axis, which provides a novel direction for the mechanism of MIRI.

## 2 MATERIAL AND METHODS

### 2.1 Cell culture and treatment

Rat embryo‐derived cardiomyocyte line H9C2 cell was purchased from Shanghai Institute of Biological Science, CAS and cultured by 10% foetal bovine serum (Gibco), supplemented with 100 U/mL penicillin and 100 μg/mL streptomycin.

For the hypoxia/reoxygenation (H/R) model, cells were washed with hypoxic solution (made of NaCl 98.5 mM, KCl 10.0 mM, CaCl_2_ 1.8 mM, NaHCO_3_ 6.0 mM, Sodium Lactate 40 mM, HEPES 20 mM, NaH_2_PO_4_ 0.9 mM, MgSO_4_ 1.2 mM, pH 6.8 and saturated with 95% N_2_ and 5% CO_2_) and incubated in hypoxia reoxygenation (A/R) device (continuously filled with 95% N_2_ and 5% CO_2_) at 37°C for 3 hours. Then, discard the hypoxic fluid and added reoxygenation solution (NaH_2_PO_4_ 0.9 mM, NaCl 129.5 mM, MgSO_4_ 1.2 mM, KCl 5.0 mM, glucose 5.5 mM, NaHCO_3_ 20 mM, CaCl_2_ 1.8 mM, HEPES 20 mM, pH 7.4) in A/R device at 37°C for 2 hours.

### 2.2 Cell transfection

The LINC00261‐overexpression plasmids (pcDNA3.1‐LINC00261), FOXO3a‐overexpression plasmids (pcDNA3.1‐FOXO3a), NRF2‐overexpression plasmids, vector controls and miR‐23b‐3p mimics were synthesized and purchased from GenePharma. Lipofectamine 2000 (Invitrogen) was used to transfect the cells according to the manufacturer's protocol.

### 2.3 Cell viability and LDH activity measurement

Cell viability was detected by Cell Counting Kit‐8 (CCK‐8; Dojindo Laboratories). Cells with a density of 5 × 10^3^/well were seeded in 96‐well plates and incubated with 10 μl of CCK‐8 solution for 2 hours at 37°C. Cell viability was quantified by a microplate reader at 450 nm. For LDH activity, cell supernatants were collected and detected using a biochemical analyzer (Beckman).

### 2.4 Apoptosis assay

For myocardium apoptosis level, terminal deoxynucleotidyl transferase dUTP nick end labelling (TUNEL) assay was performed according to the Situ Cell Death Detection kit (Roche) manufacturer's protocols. Cardiac tissues were stained with 1:9 ratios TUNEL reaction mixture and incubated for 1 hours at dark. After washed three times, DAB solution developed colour, stained the nucleus and photographed under the light microscope to count the positive cells.

### 2.5 Recombinant adeno‐associated virus 9 (rAAV9) transfection and establishment of rat I/R model

The study was in accordance with the Institutional Animal Care and Use and approved by the committee of The Second Hospital of Hebei Medical University. Sixty male Sprague‐Dawley rats (6‐8 week) were purchased from Vitalriver Laboratory Animal Company. Thirty rats were divided into Sham group (n = 10) and I/R group (n = 20), the other thirty rats were divided into (adeno‐associated virus) AAV‐NC group (n = 15) and AAV‐LINC00261 group (n = 15). The AAV‐LINC00261 group was injected with a single tail vein of 15 μl PBS diluting 5 × 10^10^ particles of AAV delivering overexpressed plasmids obtained by Hanbio. The AAV‐NC group was injected with a same dose of scrambled vector as control. Three weeks after injection, the expression of LINC00261 in the myocardium was detected and I/R model was established for further experiments.

Rats with or without transfected with adenovirus were anaesthetized with 2% pentobarbital sodium and intubated, contacted with a standard limb lead II electrocardiogram (ECG) and small animal ventilator. The heart was exposed through thoracotomy and pericardiotomy, and the left anterior descending coronary artery was ligated to the distal myocardial pale with live ligation. After the ligation for 30 minutes, the suture was released and reperfusion was performed for 120 minutes.

### 2.6 Microarray analysis

The fresh myocardial tissues from Sham group and I/R group were obtained, and total RNA was extracted using Trizol reagent. After quantitative analysis by the NanoDrop ND‐1000, total RNA was reverse transcribed into double‐stranded cDNA, and cRNA is synthesized for second round of reverse transcription into cDNA. Then, samples were labelled and fragmented for Affymetrix Human OElncRNA hybridization. Affymetrix Scanner 3000 and Affymetrix GeneChIP Command Console software(version 4.0) were used for analyse the original data.

### 2.7 Haemodynamic parameters

After 120 minutes of reperfusion, the rats were anaesthetized, immobilized and bilateral common carotid arteries were separated. The right common carotid artery was intubated to the left ventricle, and the left ventricular systolic pressure (LVSP), left ventricular end‐diastolic pressure (LVEDP), left ventricular maximum ascending rate and ascending rate (±dp/dt_max_) were measured using adopts 8‐channel physiological signal acquisition and processing system.

### 2.8 Measurement of infarct size

Heart was weighted and cut into four pieces horizontal slices from the apex to the base. The slices were immersed with 0.5 mg/ml NBT at 37°C for 10 minutes to achieve complete staining. The infarct size was calculated as the weight of ischemic myocardium zone/the weight of left ventricular myocardium.

### 2.9 Myocardial enzymes assay

Blood was taken from the abdominal aorta of rats, serum was separated, and the levels of LDH and CK‐MB were detected according to the instructions of corresponding kits.

### 2.10 Quantitative real‐time polymerase chain reaction (qRT‐PCR)

Total RNAs from myocardial tissues or cell line were isolated using Trizol reagent (Invitrogen). cDNA was synthesized with specific stem‐loop primers by the isolated RNA using a Reverse Transcriptase kit (Takara). Quantitative real‐time polymerase chain reaction (qRT‐PCR) was performed using Power SYBR green (Takara). The relative expression of genes was normalized to GAPDH and expressed as fold change compared with internal standard with 2^−ΔΔCT^ method. The primers sequences were shown in Table 1.

**Table 1:** The primer sequences of the related genes

Gene	Sequence of primers (5'‐3')
‐63500‐889100LINC00261	Forward: 5'‐ACATTTGGTAGCCCGTGGAG‐3' Reverse: 5'‐TCTTCCCCGGAGAACTAGCA‐3'
miR‐23b‐3p	Forward: 5'‐ACACTCCAGCTGGGATCACATTGCCAGGGAT‐3' Reverse: 5'‐CTCAACTGGTGTCGTGGAGCGAGGTGGTAAT‐3'
NRF2	Forward: 5'‐CTTGGCCTCAGTGATTCTGAAGTG‐3' Reverse: 5'‐CCTGAGATGGTGACAAGGGTTGTA'
FOXO3a	Forward: 5'‐CGGACAAACGGCTCACTCT‐3' Reverse: 5'‐GGACCCGCATGAATCGACTAT‐3'
GAPDH	Forward: 5'‐AGAAGGCTGGGGCTCATTTG‐3' Reverse: 5'‐AGGGGCCATCCACAGTCTTC‐3'
U6	Forward: 5'‐GCTTCGGCAGCACATATACTAAAAT‐3' Reverse: 5'‐CGCTTCACGAATTTGCGTGTCAT‐3'
β‐actin	Forward: 5'‐CTGGGACGACATGGAGAAAA‐3' Reverse: 5'‐AAGGAAGGCTGGAAGAGTGC‐3'

### 2.11 Western blotting assay

Tissues or cellular protein were extracted using lysis buffer (Solarbio). The lysates were run on 12% sodium dodecyl sulphate‐polyacrylamide gel electrophoresis (SDS‐PAGE) and transferred to polyvinylidene difluoride (PVDF) membranes (Millipore). Then, the bands were incubated with primary antibodies at 4°C overnight: anti‐Bax (1:1000, 2772; Cell Signalling Technology), anti‐Bcl2 (1:1000, 3498; Cell Signalling Technology), cleaved caspase 3 (1:1000, 9661; Cell Signalling Technology) and GAPDH (1:1000, 5174; Cell Signalling Technology). After washed by TBST, the bands were incubated with horseradish peroxidase‐labelled secondary antibody at room temperature for 2 hours and detected by an enhanced chemiluminescence detection system (Bio‐Rad) according to the manufacturer's protocol.

### 2.12 Luciferase reporter assay

Combining NCBI‐Gene and starBase to find the complementary binding region sequence of the target gene promoter region and miR‐23b‐3p, and design the corresponding mutant sequence. Sequence of LINC00261 and NRF2 3'‐UTR was amplified using RT‐PCR and inserted into the pGL3 luciferase vector (Promega). The primer of sequence of NRF2 mRNA 3'‐UTR was 5'‐CTTCGTGCTGGAAGATGACTCCT‐3' (forward), 5'‐CTAAGGCCAAGGCCTTAAGGTGA‐3' (reverse). HEK293 cells were transfected with firefly luciferase vector (100 ng) and Renilla luciferase expression vector (10 ng). Cells were transiently cotransfected with luciferase‐reporter plasmids (Promega) with Lipofectamine 2000 according to the manufacturer's instructions. After cotransfection with indicated plasmids for 48 hours, Dual‐Luciferase Reporter Assay System (Promega) was followed. Renilla luciferase (pRL‐TK) acted as an internal control to normalize transfection efficiencies.

### 2.13 Subcellular fractionation location

The distribution of LINC00261 in the nuclear and cytoplasmic fractions of H9C2 cell was determined with the PARIS kit (Ambion) according to manufactures’ instructions.

### 2.14 Chromatin immunoprecipitation (ChIP) assay

The ChIP assays were performed using EZ‐CHIP kit (Millipore). Briefly, H9C2 cells were cross‐linked with 1% formaldehyde at RT for 10 minutes and quenched in 125 mM glycine for 5 minutes. The cross‐linked chromatin was detected by ultrasound to obtain DNA fragments with an average length of 0.5‐2 kb. The antibody anti‐FOXO3a (ab12162; Abcam) and normal rabbit IgG (ab172730; Abcam) were utilized to precipitate the cross‐linked protein‐DNA complexes. qPCR was used to analyse the precipitated DNA.

### 2.15 RNA immunoprecipitation (RIP) assay

RIP experiments were performed using a Magna RIP RNA‐Binding Protein Immunoprecipitation Kit (Millipore) according to the manufacturer's instructions. Cells were lysed using complete RIP lysis buffer containing RNase Inhibitor (Millipore) and protease inhibitor. Different cell lysis solutions were incubated with the RIP buffer containing magnetic beads coated with anti‐human argonaute 2 (Ago2) antibodies (Millipore). IgG (Millipore) acts as a negative control (input group). Lysates were immunoprecipitated with beads at 4°C overnight. RNA was purified from RNA protein, reverse transcribed by PrimeScript 1st Strand cDNA Synthesis Kit (Takara), and then detected by RT‐PCR.

### 2.16 Statistics analysis

The data were expressed as mean ± standard deviation or Interquartile range. GraphPad Prism 7.0 (GraphPad Software) was used to perform the statistical analysis and graph plotting. One‐way ANOVA and Student's *t* test were used for different comparison between multiple group and two groups. *P*<.005 or *P*<.001 was considered as a mark of statistically significant.

## 3 RESULTS

### 3.1 LINC00261 expression is down‐regulated in I/R myocardial tissues and H/R cardiomyocyte

**Figure 1:**
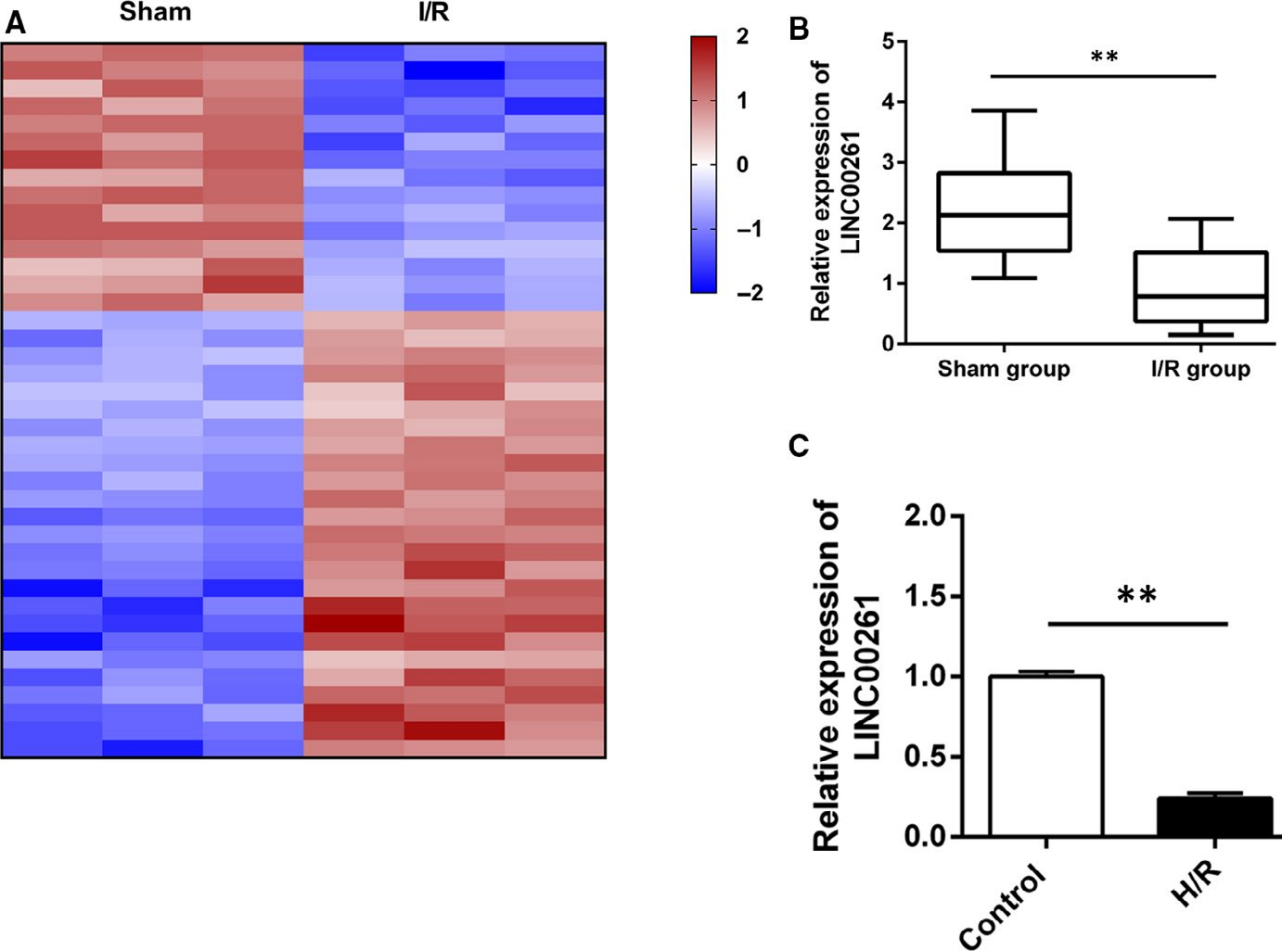
LncRNAs expression profiles in I/R myocardium model exposed an significantly down‐regulated lncRNA, LINC00261. (A) Heatmaps show the differentially expressed transcripts in rats I/R group and sham group. Blue represents a lower fold‐change and red represents a higher fold‐change, n = 3. (B) qRT‐PCR verified the low expression level of LINC00261 in I/R myocardium, n = 7. (C) Relative expression of LINC00261 in H/R H9C2 cell **P＜0.01, n = 3

To explore differentially expressed lncRNAs in I/R myocardial tissues, we used rat microarray analysis to screen the expression profiles. The chip data were proposed by GeneSpring software (version 14.0) software to analyse the differential genes between two groups. The screening criteria were up or down two times, and the *P* value of the *t* test was <0.05. Compared with sham group, a total of 254 lncRNAs were up‐regulated and 145 lncRNAs were down‐regulated in I/R group (Figure 1A). We took the fivefold reduction as the limit and obtained 4 differential lncRNAs. After PCR verification, LINC00261 showed the most significant reduction and high reproducibility. Interestingly, LINC00261 was obviously down‐regulated in I/R myocardial tissues and H/R cardiomyocyte (Figure 1B,C).

### 3.2 Overexpression of LINC00261 promotes cardiac function and reduces myocardium apoptosis

**Figure 2:**
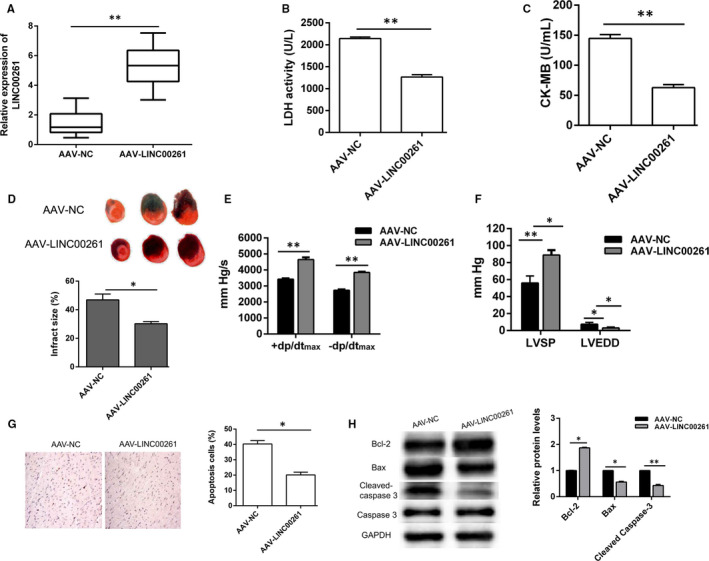
Overexpression of LINC00261 improves cardiac function and inhibits apoptosis. (A) Adenovirus was transfected into myocardial tissues of rats, and the overexpression efficiency of LINC00261 was detected by qRT‐PCR, n = 8. (B) LDH content and (C) CK‐MB concentration in rat serum were detected using the corresponding kits, n = 8. (D) NBT staining and calculation of myocardial infarction area, n = 3. (E, F) Multi‐channel physiological signal acquisition and processing system were used for detecting LVSP, LVEDD and ± dp/dt_max_, n = 8. (G) TUNEL staining was performed to detect apoptosis rate. *P < .05, **P < .01, n = 3. (H) The relative protein expression of Bcl2, Bax, cleaved caspase 3 and caspase 3 was detected by Western blotting, relative protein expression were shown with histogram, n = 3

An adeno‐associated virus‐carrying rat LINC00261gene was constructed and packaged in HEK293 cells. After collection and purification, the virus was injected into the rat via the tail vein. Three weeks later, the transfection efficiency and expression of LINC00261 in the myocardium was detected by qRT‐PCR **(**Figure S1). As shown in Figure 2A, the expression of LINC00261 was significantly higher in AAV‐LINC00261 group compared with AAV‐NC group. Functional test results showed that overexpression of LINC00261 significantly reduces I/R‐induced increasing of serum myocardial enzymes LDH and CK‐MB (Figure 2B,C). Compared with AAV‐NC group, overexpression of LINC00261 significantly reduced myocardial infarct size in rats (Figure 2D). Haemodynamic parameter results indicated that left ventricular function was remarkably recovered in AAV‐LINC00261 group (Figure 2E,F). TNUEL assay and Western blot results showed that myocardium apoptosis significantly decreased in AAV‐LINC00261 group (Figure 2G,H). These data suggest that overexpression of LINC00261 significantly reduces rats MIRI.

### 3.3 Overexpression of LINC00261 reduces H/R induced cardiomyocytes apoptosis

**Figure 3:**
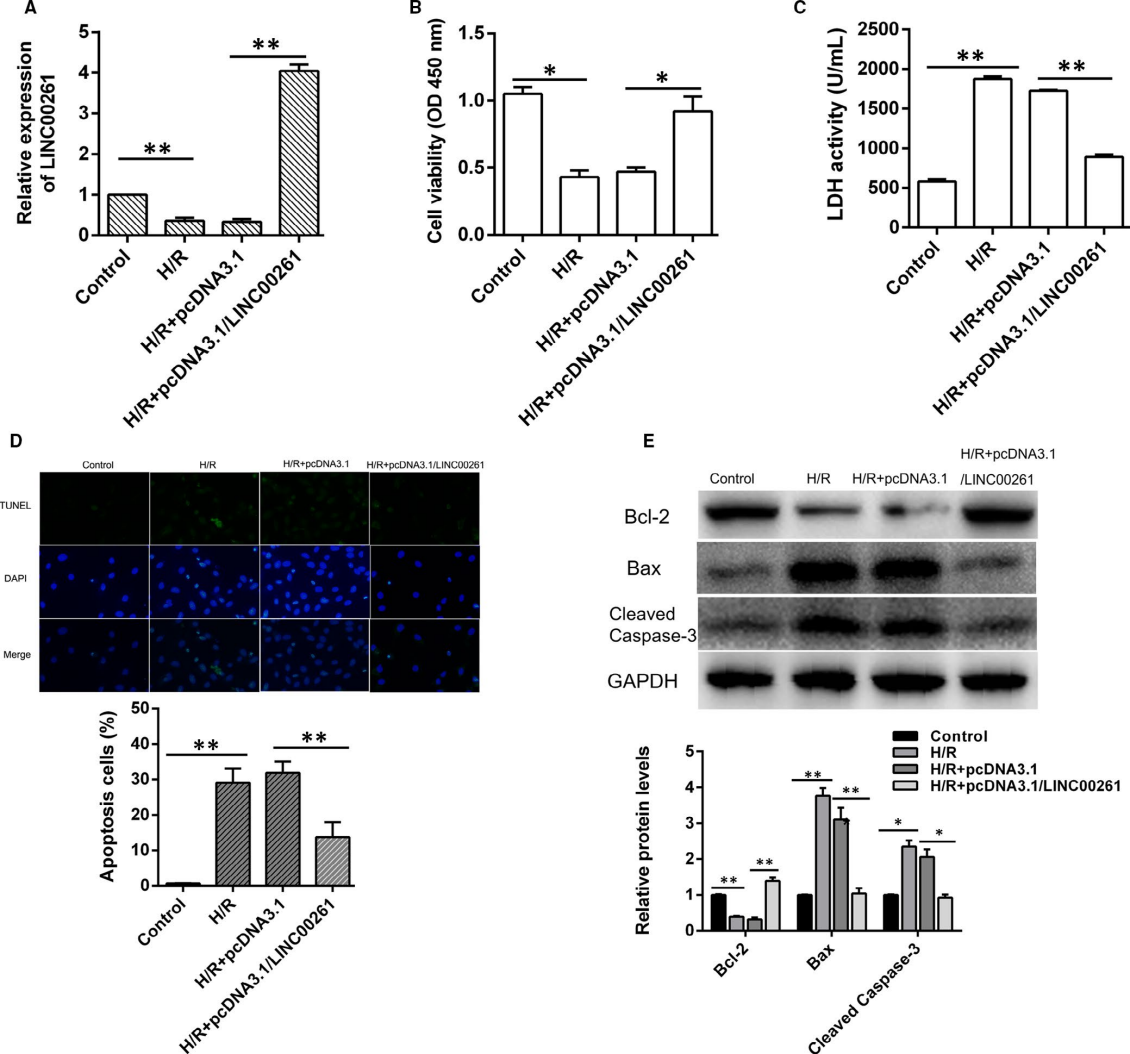
Overexpression of LINC00261 significantly alleviates H/R‐induced cardiomyocytes apoptosis, n = 3. (A) Relative expression of LINC00261 was detected by qRT‐PCR, n = 3. (B) Cell viability and (C) LDH content were measured by spectrophotometry, n = 3. (D) Cell apoptosis was detected by TUNEL stain. (E) Bcl‐2, Bax and Cleaved Caspase‐3 protein expression was detected by Western blotting. *P < .05, **P < .01, n = 3

To further understand the effects of LINC00261 on H/R induced cardiomyocytes injury, rat embryonic cell line H9C2 cell was cultured and transfected with pcDNA3.1/LINC00261 or pcDNA3.1 vector (Figure S2). qRT‐PCR results indicated that LINC00261 was significantly up‐regulated in pcDNA3.1/LINC00261 group (Figure 3A). Cell viability and LDH activity were measured and the results showed that LINC00261 overexpression reduced H9C2 cell damage (Figure 3B,C). Moreover, LINC00261 overexpression reduced H/R induced increase of cell apoptosis (Figure 3D). In consistence, Western blot results showed that Bax and cleaved caspase 3 expression were decreased and Bcl2 expression was increased when cell transfected with pcDNA3.1/LINC00261 (Figure 3E). These results indicate that LINC00261 overexpression of LINC00261 has protective effect against cardiomyocyte apoptosis.

### 3.4 LINC00261 positively regulates NRF2 by sponging miR‐23b‐3p in cardiomyocytes

**Figure 4:**
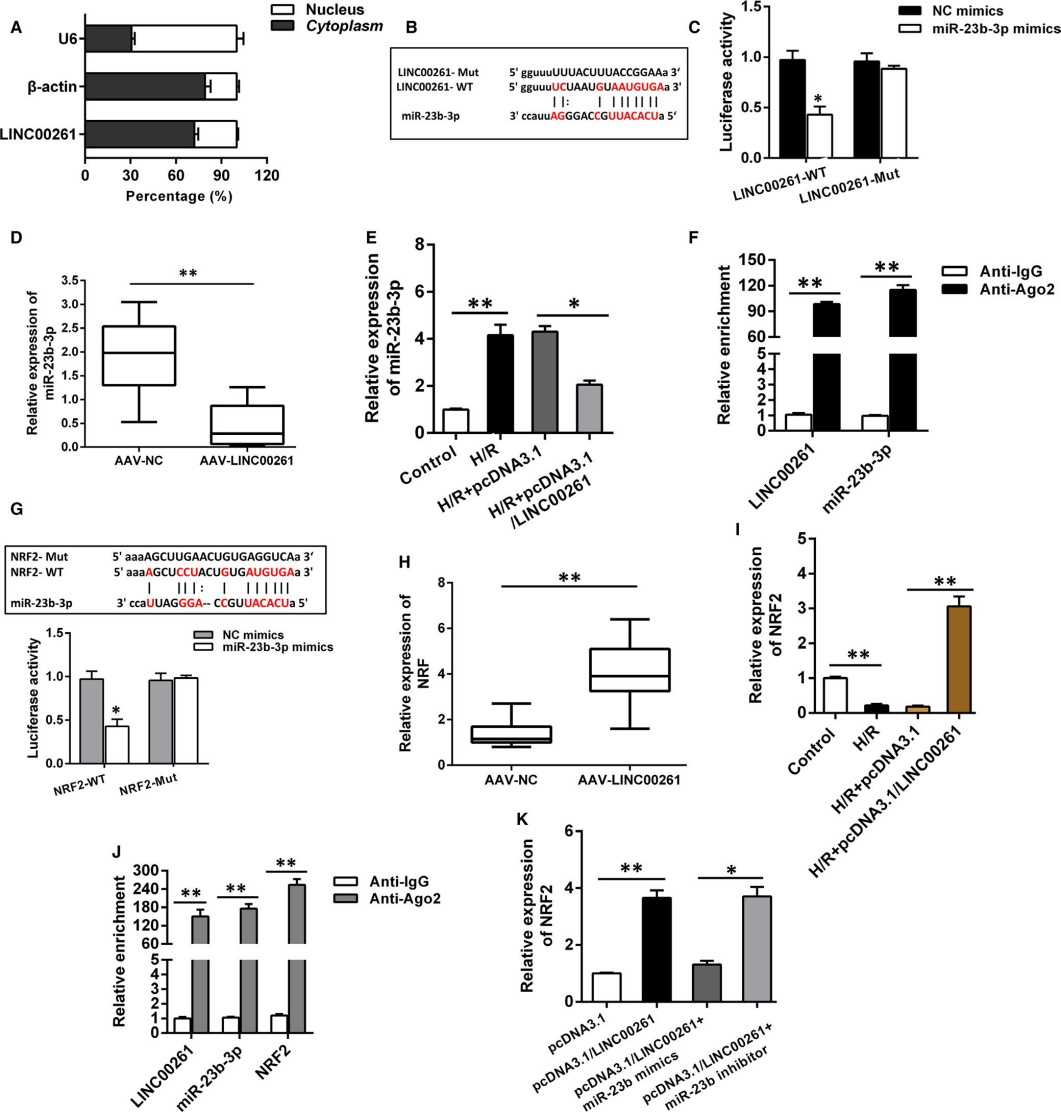
LINC00261 regulated NRF2 by directly target miR‐23b‐3p in H9C2 cell. (A) Subcellular location of LINC00261 in H9C2 cell was detected via subcellular fractionation, n = 3. (B) Bioinformatics tools (http://starbase.sysu.edu.cn/) indicated the direct binding within miR‐23b‐3p and LINC00261 3'‐UTR. (C) Cells with miR‐23b‐3p mimics or negative control were cotransfected with LINC00261‐WT or LINC00261‐Mut and luciferase activity was measured by luciferase assay, n = 3. (D) The expression of miR‐23b‐3p in myocardium was detected by qRT‐PCR, n = 3. (E) Cell was transfected with pcDNA3.1 or pcDNA3.1/LINC00261, and miR‐23b‐3p expression was detected by qRT‐PCR, n = 3. (F) RIP assay showed the binding ability between LINC00261 and miR‐23b‐3p, n = 3. (G) Bioinformatics tools (http://starbase.sysu.edu.cn/) indicated the direct binding within miR‐23b‐3p and NRF2 3'‐UTR. Luciferase reporter assay confirmed the binding with the miR‐23b‐3p and wild‐type of NRF2 3'‐UTR. (H) Relative expression of NRF2 in myocardium was detected by qRT‐PCR, n = 3. (I) Cell was transfected with pcDNA3.1 vector or pcDNA3.1/LINC00261, and NRF2 expression was detected by qRT‐PCR, n = 3. (J) RIP assay was performed to detect the binding ability between LINC00261, miR‐23b‐3p and NRF2 in H9C2 cell, n = 3. (K) The expression of NRF2 in H9C2 cell transfected with different plasmids was detected by qRT‐PCR. *P < .05, **P < .01, n = 3

The important mechanism of lncRNA post‐transcriptional regulation is to regulate the expression of miRNAs in the cytoplasm through competitive endogenous RNAs (ceRNAs).^14^ We isolated the cytoplasm and nucleus and detected the expression of LINC00261 by qRT‐PCR. The results showed that LINC00261 was mainly expressed in cytoplasm (Figure 4A). Bioinformatics analysis predicted that there are binding sites between miR‐23b‐3p and 3'‐UTR of LINC00261 (Figure 4B). Luciferase reporter assay showed that overexpression of miR‐23b‐3p inhibited the luciferase activity of reporter vectors containing wild‐type 3'‐UTR of LINC00261 (Figure 4C). Moreover, overexpression of LINC00261 significantly inhibited expression of miR‐23b‐3p in I/R myocardial tissues and H/R induced H9C2 cell(Figure 4D,E). Furthermore, the RIP assay showed that miR‐23b‐3p mainly bound to the region of LINC00261 (Figure 4F). To continue, we screened the potential mRNAs that could be targeted by miR‐23b‐3p. Similarly, based on the predicted sites and luciferase reporter results, miR‐23b‐3p overexpression inhibited the luciferase activity of the wild‐type reporter vector, but not the mutant one (Figure 4G), while LINC00261 overexpression remarkably increased NRF2 level in myocardial tissues and cell line (Figure 4H,I). In addition, RIP assay showed the higher enrichment of LINC00261, miR‐23b‐3p and NRF2 in Ago immunoprecipitations compared with control IgG immunoprecipitations (Figure 4J). qRT‐PCR results further proved the regulating relationship between LINC00261, miR‐23b‐3p and NRF2 (Figure 4K). Above results suggest that LINC00261 regulating NRF2 by sponging miR‐23b‐3p in cardiomyocytes.

### 3.5 LINC00261 regulates cell apoptosis of cardiomyocytes via miR‐23b‐3p/NRF2 axis

**Figure 5:**
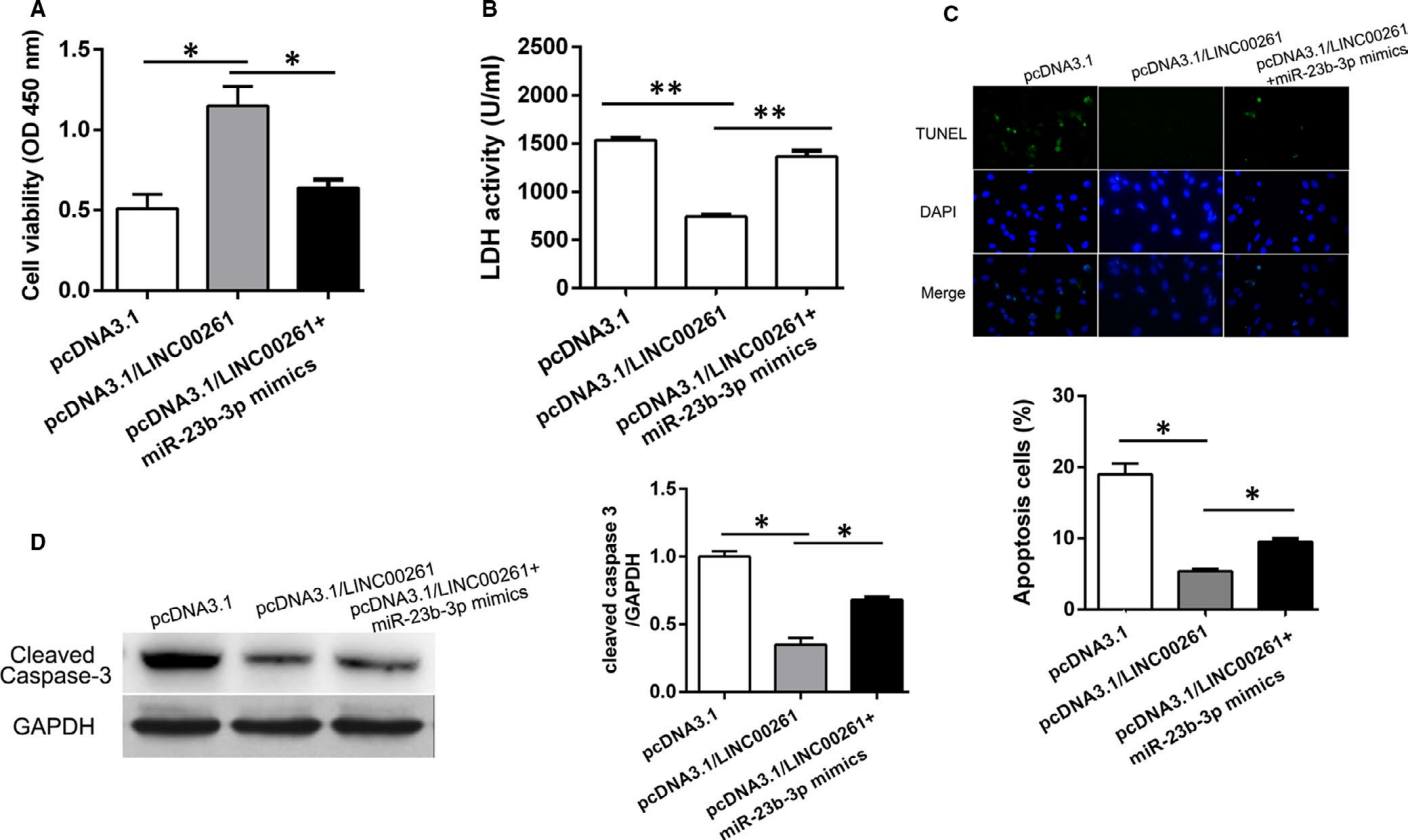
LINC00261 inhibits cardiomyocytes apoptosis via miR‐23b‐3p/FOXO3a axis. (A) CCK‐8 assay was conducted to detect cell viability in different groups, n = 3. (B) LDH activity (C) Cell apoptosis rate was evaluated by TUNEL assay, n = 3. (D) The expression of cleaved caspase‐3 was detected by Western blotting. *P < .05, n = 3

To further confirm the role of LINC00261/miR‐23b‐3p/NRF2 axis in MIRI, rescue experiments were performed, respectively. H9C2 cells with different transfection treatments and then induced with hypoxia and reoxygenation. As shown in Figure 5A,B, cotransfection of pcDNA3.1/LINC00261 and miR‐23b‐3p mimics reversed the increase in cell viability due to LINC00261 overexpression. Moreover, TUNEL assay and Western blot indicated that LINC00261 overexpression inhibits cell apoptosis, while cotransfection pcDNA3.1/LINC00261 and miR‐23b‐3p mimics rescued the effect of pcDNA3.1/LINC00261 (Figure 5C,D). These results LINC00261 regulates cell apoptosis of cardiomyocytes via miR‐362‐3p/NRF2 axis.

### 3.6 Transcription factor FOXO3a promotes LINC00261 transcription

**Figure 6:**
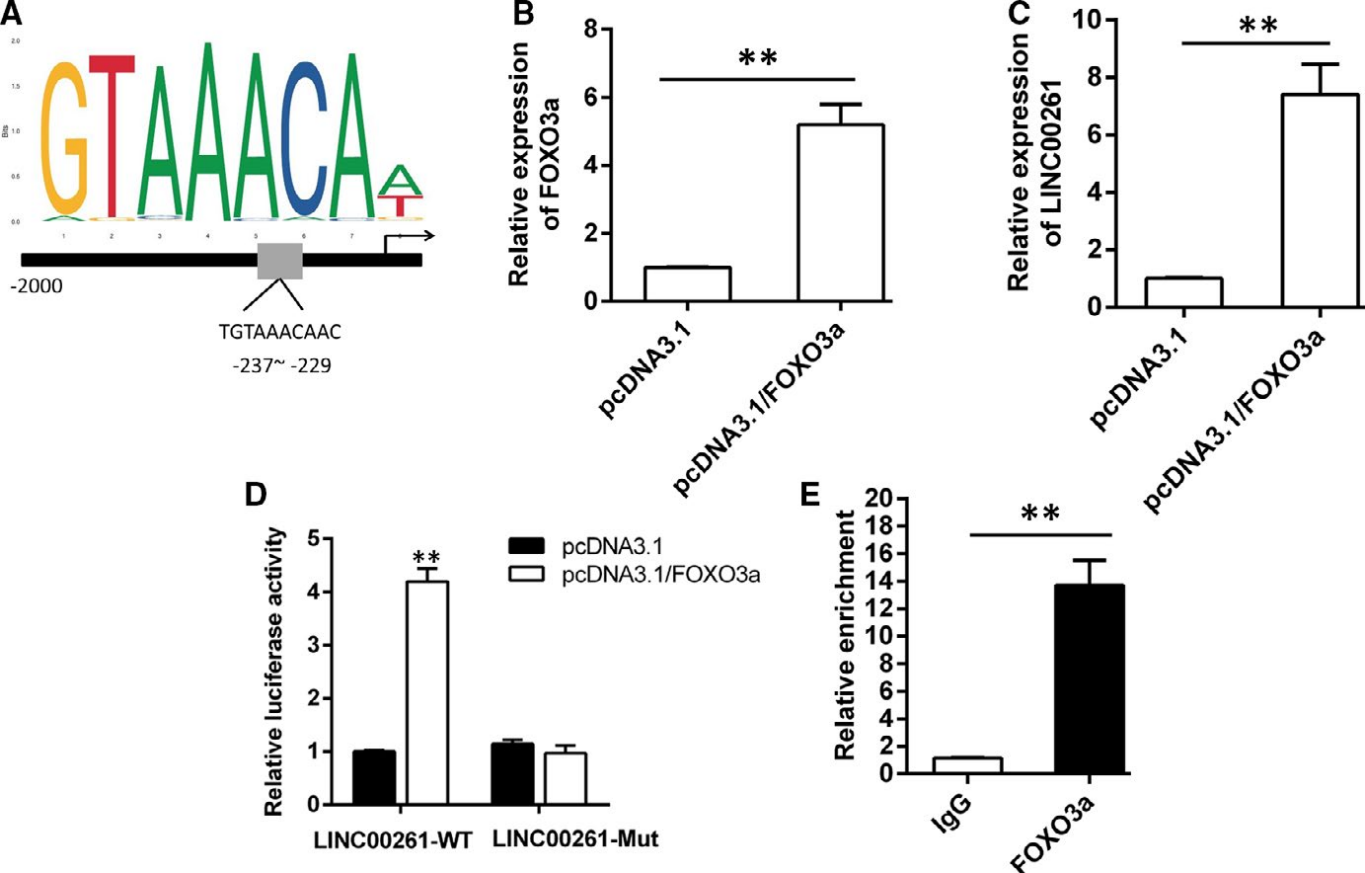
FOXO3a stimulated LINC00261 transcription in H/R‐induced cardiomyocyte. (A) The predicted putative FOXO3a‐binding sites in human LINC00261 promoter from JASPAR database. (B) The overexpression efficiency of FOXO3a was validated by qRT‐PCR, n = 3. (C) The expression of LINC00261 was detected when cells transfected with pcDNA3.1/LINC00261 or pcDNA3.1, n = 3. (D) The luciferase reporter plasmids containing the wild‐type 3'‐UTR region or mutant 3'‐UTR region of LINC00261 were cotransfected into cells with FOXO3a or vector. *P < .05, **P < .01, n = 3. (E) CHIP assay was performed to detect the binding capacity between FOXO3a and LINC00261 promoter in H9C2 cell, n = 3

Studies show that lncRNA acts as a ligand and combines with some transcription factors to form a complex to control gene transcription activity.^15^, ^16^ By use of JASPAR database (http://jaspar.genereg.net/ ), transcription factor FOXO3a was predicted to potentially bind to LINC00261 promoter (Figure 6A). Cells were transfected with pcDNA3.1/FOXO3a or pcDNA3.1 in cardiomyocyte, and the transfection efficiency of FOXO3a was verified by qRT‐PCR (Figure S3). The impact of FOXO3a on LINC00261 was investigated (Figure 6B). As shown in Figure 6C, FOXO3a overexpression significantly promotes LINC00261 expression. The luciferase reporter assay results showed that FOXO3a overexpression significantly increases the luciferase activity of LINC00261 in H9C2 cell (Figure 6D). Moreover, CHIP assay results directly confirmed that FOXO3could binds to the promoter region of LINC00261 (Figure 6E). These results indicate that FOXO3a could promote LINC00261 transcription and up‐regulation its expression in H9C2 cell.

## 4 DISCUSSION

Accumulating studies have confirmed that lncRNAs are involved in the pathological process of MIRI. For instance, lncRNA GAS5 knockdown suppresses MIRI via the miR‐335/ROCK1/AKT/GSK‐3β axis.^17^ LncRNA H19/miR‐877‐3p/Bcl‐2 pathway has been identified involved in regulation of mitochondrial apoptosis during MIRI.^18^ Not only acts as miRNAs, lncRNA also participates in cardiomyocyte autophagy, apoptosis and necrosis by regulating downstream targets.^19^ In addition, the characteristics of tissue specificity and stable expression make lncRNAs promising as biomarkers for disease diagnosis. Some of these lncRNAs have been used in the diagnosis of coronary heart disease and acute myocardial infarction.^20^ In this study, we used microarray analysis identified that LINC00261 expression was significantly down‐regulated in rat I/R myocardium and H/R‐induced H9C2 cell line. Overexpression of LINC00261 with adenovirus significantly improves cardiac function and reduces myocardial apoptosis. Similarly, H9C2 cell line overexpressed LINC00261 obviously reduced apoptosis. These data support that LINC00261 is a protective molecule for myocardial I/R, and its role is exerted by reducing apoptosis.

Known functions of lncRNAs include regulation of transcription, binding proteins and microRNAs sponges are closely related to the occurrence and development of various cardiovascular diseases. There are a large number of miRNAs binding sites on lncRNAs, which are specifically combined with a certain sequence of the target miRNA by means of base complementation, thus acting as a miRNAs sponge. MiR‐23b plays a crucial role in differentiation of multiple cells, including keratinocytes, tumour cells, chondrocyte et al In lens epithelial cells, miR‐23b‐3p can regulate apoptosis and autophagy by targeting silent SIRT1 under oxidative stress.^21^ Microarray analysis of miRNAs expression in coronary sinus blood from patients with heart failure showed that miR‐23b‐3p expression was significantly increased, indicating that it is involved in the progression of heart failure.^22^ In our data, miR‐23b‐3p was significantly up‐regulated in I/R myocardial tissues and H/R‐induced H9C2 cell. Based on the prediction of biological information analysis, luciferase report assay, RIP assay and qRT‐PCR, we determined the targeting relationship between miR‐23b‐3p and LINC00261, and identified NRF2 as a downstream target of miR‐23b‐3p by the similar methods. Thus, the regulatory axis of LINC00261 on cardiomyocytes apoptosis under H/R conditions was clarified.

Recent studies have shown that transcription factors can not only regulate the encoded proteins, but regulate the transcription of lncRNAs.^23^ FOXO3a has been shown to bind to the promoter of lncRNA CASC11, thereby promoting the tumorigenesis of non‐small cell lung cancer.^24^ In mice I/R model, the activation of FOXO3a has the protective effect of initiating antioxidant gene expression and reducing myocardial damage.^25^ Our results demonstrate that FOXO3a can bind to the LINC00261 promoter and enhance its expression, thereby promoting the increase of NRF2 expression and exerting the role of anti‐oxidative stress to reduce cell apoptosis.

In summary, our study explored the function and mechanism of lncRNA LINC00261 screened by microarray analysis. The results demonstrated that LINC00261 was both down‐regulated in I/R myocardial tissues and H/R‐induced cardiomyocyte. LINC00261 overexpression significantly reduces cell apoptosis. Further results indicated that transcription factor FOXO3a‐mediated LINC00261 inhibits cardiomyocytes apoptosis by sponging miR‐23b‐3p to maintaining NRF2 transcription.

## CONFLICT OF INTEREST

The authors declare that there are no conflicts of interests.

## **AUTHORS** **CONTRIBUTIONS**

Ruining Zhang, Yongjun Li and Xiaopeng Liu performed the research; Suyun Liu, Shan Qin and Bingyan Guo designed the research contents; Liang Chang and Liu Huang analysed the data; Ruining Zhang and Suyun Liu wrote the paper.

## Supporting information

Supplementary Material

## Data Availability

The data that support the findings of this study are not available.
