# Supplementary material for: FOXO3a‐mediated long non‐coding RNA LINC00261 resists cardiomyocyte hypoxia/reoxygenation injury via targeting miR23b‐3p/NRF2 axis
Source: J Cell Mol Med. 2020 Jun 18;24(15):8368–78. doi: 10.1111/jcmm.15292 (PMC7412708; doi:10.1111/jcmm.15292)

1. Myocardial tissue transfection efficiency

Under the fluorescence microscope, no green fluorescence was observed in the AAV-NC group, while in the AAV-LINC00261 group, a significantly evenly distributed green fluorescence was seen in the myocardium at 1 week and 3 week after transfection.


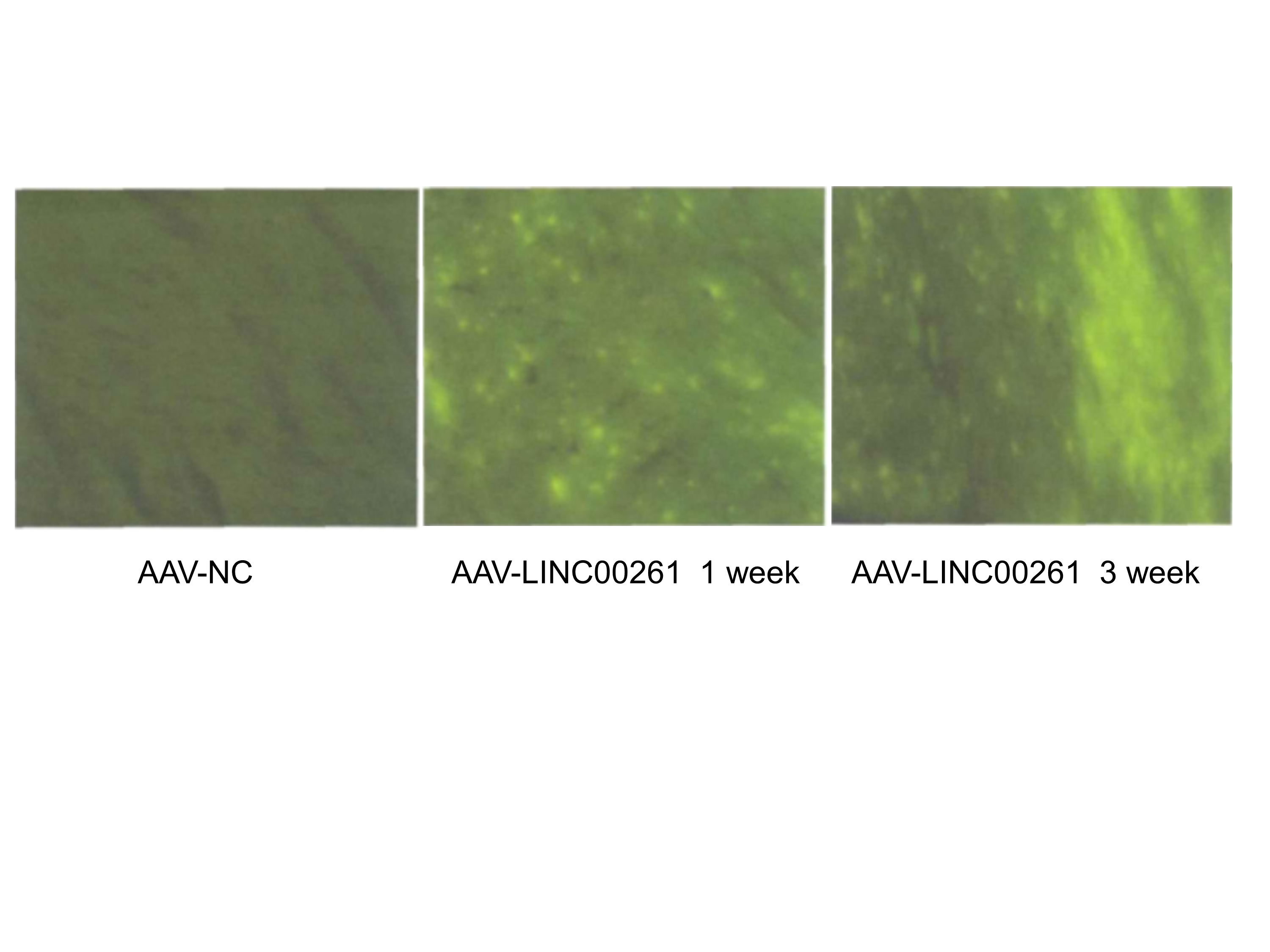


Figure 1. Adenovirus transfection fluorescence intensity (×200).

2. The LINC00261 transfection effect in H9C2 cells


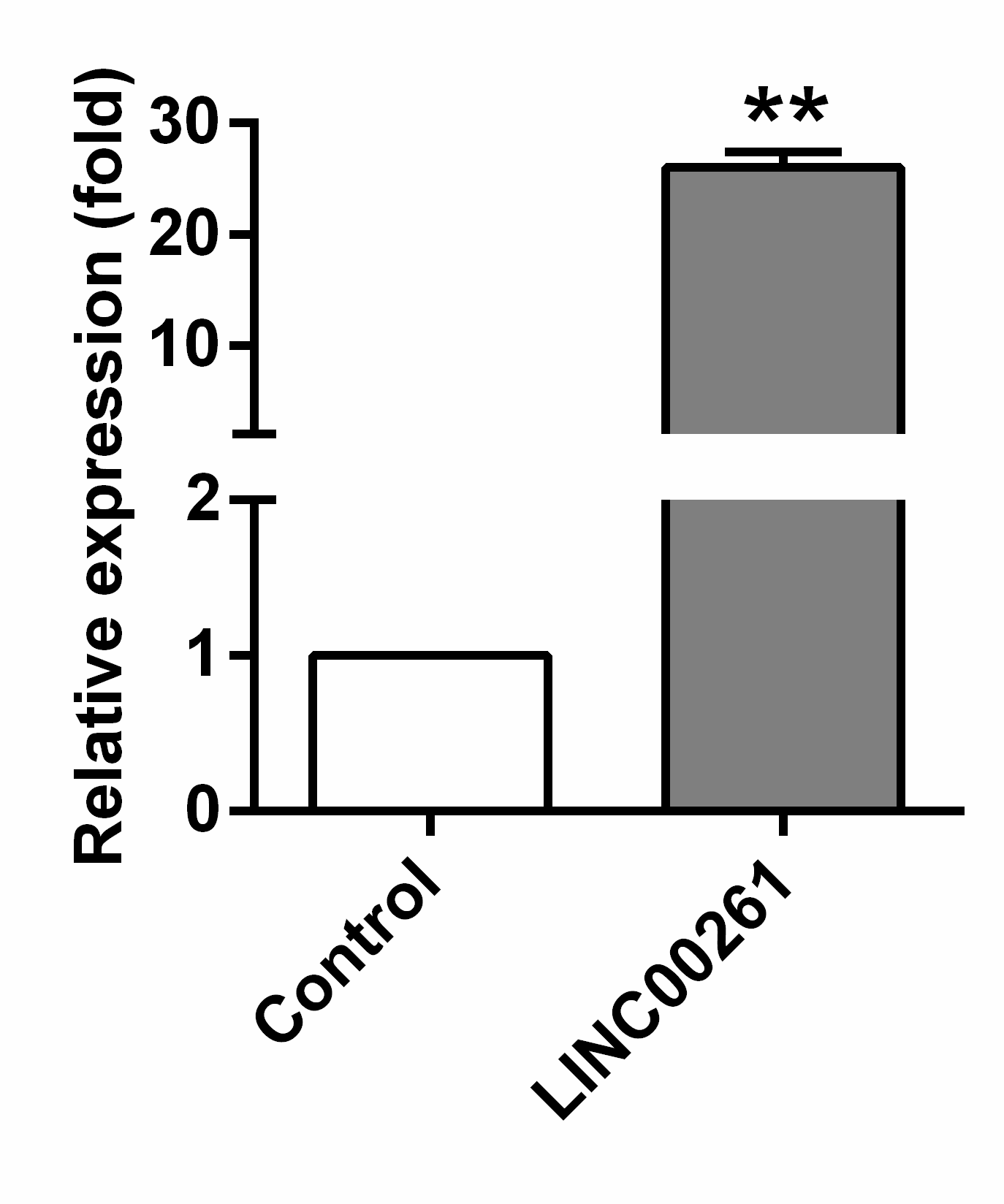


3. The FOXO3a transfection effect in H9C2 cells


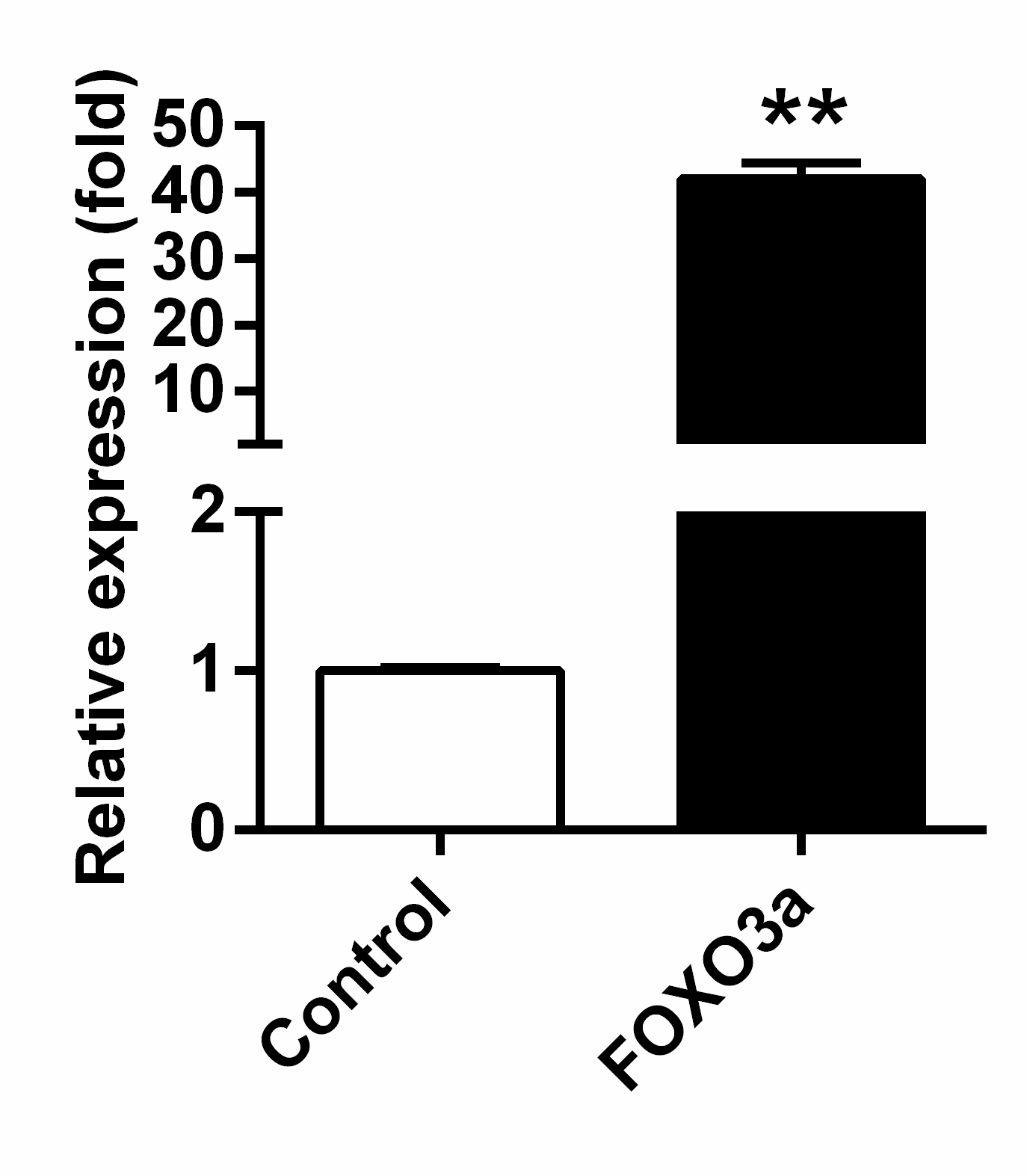

Supplement: Supplementary file 1 — Supplementary Material [file JCMM-24-8368-s001.docx]
